# Clinical Application of Autologous Adipose Stem Cells in Patients with Multiple Sclerosis: Preliminary Results

**DOI:** 10.1155/2016/5302120

**Published:** 2016-09-28

**Authors:** Adam Stepien, Natalia L. Dabrowska, Marzena Maciagowska, Renata Piusinska Macoch, Aleksandra Zolocinska, Slawomir Mazur, Katarzyna Siennicka, Emilia Frankowska, Rafał Kidzinski, Małgorzata Chalimoniuk, Zygmunt Pojda

**Affiliations:** ^1^Department of Neurology, Military Institute of Medicine, Warsaw, Poland; ^2^Laboratory of Cellular Engineering, M. Skłodowska-Curie Memorial Cancer Center and Institute of Oncology, 02-781 Warsaw, Poland; ^3^Department of Breast Cancer and Reconstructive Surgery, Oncology Center-Institute, Warsaw, Poland; ^4^Department of Radiology, Military Institute of Medicine, Warsaw, Poland; ^5^Polish Academy of Sciences, Warsaw, Poland

## Abstract

The clinical outcome of autologous adipose stem cell (ASC) treatment of patients with multiple sclerosis (MS) was investigated following one year of observation.* Methods*. The clinical and MRI outcomes of 16 ASC-treated patients with RRMS and SPMS are reported after a one-year follow-up period.* Results.* At 18 months of follow-up, some patients showed “enticing” improvements on some exploratory efficacy measures, although a significant benefit was not observed for any measure across the entire group. Neither the progression of disability nor relapses were observed in any cases. In four patients, we found new gadolinium+ (Gd+) lesions on MRI. Our results indicate that ASC therapy is safe and does not produce any substantial side effects. Disease progression-free survival (PFS) of 18 months was seen in all patients with RRMS and SPMS. In these patients, EDSS scores did not progress above baseline scores. Gd-enhancing lesions were observed in two cases with RRMS, but these patients did not exhibit changes in EDSS score.* Conclusion*. Intrathecal treatment with ASCs is an attractive form of therapy for patients with MS but should be reserved for cases with aggressive disease progression, for cases that are still in the inflammatory phase, and for the malignant form.

## 1. Introduction

Multiple sclerosis (MS) is a disorder of the central nervous system that affects over 500,000 Europeans. Patients are typically diagnosed in their 20s or 30s. The disorder affects three times as many women as men and is more prevalent in Northern Europe [1]. The social costs associated with MS are high due to its long duration, the early loss of productivity, the need for assistance with activities of daily living, and the use of immunomodulatory treatments. Longitudinal population-based studies have found that 50% of patients require assistance with ambulation after 15 years and that over 80% of MS patients reach a level of severe and permanent disability after 30 years [2]. MS is caused by an autoimmune reaction against self-directed myelin antigens in the central nervous system. As a result of the immune deregulation that characterizes MS, axons are irreversibly damaged, which causes clinical symptoms in patients. Clinically, the disease manifests itself as relapses of neurological disability due to dysfunction of the areas in which myelin damage occurs. MS can lead to severe and permanent disability due to the axonal damage and irreversible neurodegeneration [3]. Although the causes of MS remain unknown and there is currently no cure, over the last twenty years, a number of treatments have been developed that reduce the number of relapses and slow the progression of the disease [4]. However, new therapeutic options are needed. Advances in our understanding of the underlying pathogenesis of MS will help identify potentially promising avenues of research for novel therapies with unique mechanisms of action. Autologous mesenchymal stem cells (MSCs) have been shown to induce immunomodulatory and neuroregenerative effects and to have a neuroprotective effect in the animal model of chronic experimental autoimmune encephalomyelitis (EAE) [5, 6].

Studies in animals have shown that MSCs can, under the right conditions, mature into myelin-producing cells that counter myelin loss in MS disease models [7–10]. Adipose-derived stem cells (ASCs) are adult mesenchymal stem cells (MSCs) that reside in fat. They can be isolated through enzymatic digestion and have been widely used in clinical trials involving both autologous and allogenic models: over 400 clinical trials that include MSC transplantation procedures are currently registered (https://clinicaltrials.gov/). Although stem cell therapies for a number of conditions have been previously tested with favorable safety results, few reports of their use in MS patients are available. The goal of this study was to evaluate the safety and tolerability of intrathecal autologous transplantation of ASCs over the relatively short period of one year of observation. The secondary endpoints of relapse rate and disability progression were assessed 18 months following randomization. The goal of the study was to achieve local immunomodulation of the patient's immune system by transplantation of autologous ASCs.

## 2. Materials and Methods

After the study procedures were approved by the institutional review board, 20 patients with relapsing-remitting (RR) MS (13 patients) and secondary progressive (SP) MS (7 patients) in both groups of patients with relapses were enrolled in the study. The study enrolled patients who were diagnosed with relapsing-remitting MS according to the McDonald criteria of 2013. Patients were diagnosed with secondary progressive MS if they achieved SRD (SRD: sustained reduction in disability; SRD definition: ≥1-point decrease in EDSS (Expanded Disability Status Scale) score over 6 months for patients with an EDSS score ≥2.0) during the 6 months before the stem cell procedure and did not experience a relapse.

The ASC transplantation procedures were performed from February 2014 to February 2016. The main inclusion criterion was a one-point increase in EDSS score in the year preceding the ASC implantation, with or without new or larger gadolinium- (Gd-) enhancing lesions on MRI.

In addition to the safety endpoints, the investigators tracked several efficacy outcomes, including changes in EDSS and MS Functional Scale scores, relapses, and MRI lesion burden.

The baseline clinical characteristics of these patients are summarized in Table 1. Clinical and neurologic evaluations were carried out just before the ASC procedure (baseline), 3 months after randomization, and then every 3 months for the following 18 months.

A neurologic evaluation was completed whenever the patient complained of symptoms or signs suggestive of a relapse. The neurologic assessments were performed by the same 2 neurologists. Patients whose EDSS scores were stable over the 24 months who did not relapse were considered responders. Progression was defined as an increase in EDSS score of 1 or more points.

### 2.1. Collection and Isolation of SVF Containing ASCs

Adipose tissue was collected from the patients' abdominal and femoral regions using Coleman's technique [9] and immediately transported to the stem cell laboratory. The fat tissue was mixed with buffered physiological salt solution (PBS) in a 2 : 1 vol/vol ratio and vigorously shaken. Following fat phase separation, the red cell-containing PBS was discarded, and the “fat washing” process was repeated 3x. Subsequently, 0.075% collagenase in PBS was added to the adipose tissue (1 : 2 vol/vol), and the mixture was incubated for 1.5 h at 37°C. Every 15 min, the collagenase/fat mixture was mixed by shaking for 15 sec. After the incubation period, human albumin (20% solution, 2% final concentration) was added, and the mixture was centrifuged (400 ×g, 10 min). The liquid fat and salt interphases were discarded, and the resulting cell suspension (3 mL) was filtered through a 100 *μ*m nylon mesh filter 3x. The cell suspension was washed 3x with PBS, and the final cell suspension was made in 5 mL of physiological salt (clinical grade) supplemented with 2% human albumin. Control tests included cell number and viability, sterility control, and flow cytometry characteristics of cell surface markers.

All procedures were performed in the Stem Cells and Tissue Bank, which is accredited for the processing and storage of ASCs for clinical purposes.

ASC-containing SVF was injected intrathecally (12 × 10^6^ cells/dose) at the time of enrollment, and the injection was repeated at the 3rd and 6th months using cryopreserved ASCs. The follow-up observation time varied from 12 to 16 months, and the efficacy parameters (EDSS and MS Functional Scale scores, relapse incidents, MRI lesion burden, and whole brain and gray matter atrophy rates) were monitored throughout the 12- to 16-month period.

### 2.2. MRI 

The MRI analyses included the cumulative number of new T2 lesions that appeared over the first year after therapy as well as the time of appearance of the first new T2 MRI lesion. MRI examinations were performed in a participating center with MRI scanners operating at 3 T using a standardized protocol. Contrast agent-enhanced T1-weighted images were obtained for all patients after IV injection of 0.1 mmol/kg body weight Gd-based contrast agent. MRI of the brain was obtained at screening, at baseline, and after 12 months.

The tests were performed in accordance with the Declaration of Helsinki, and the study protocol was approved by the Bioethical Committee of the Military Medical Institute in Warsaw.

## 3. Results

From 2014 to 2015, 20 patients were recruited. All of the patients completed the follow-ups at 12 and 18 months, and some of them also completed a 24-month follow-up. Among the recruited patients, 13 were in the RR phase (group I), while 7 were in the SP phase of the disease with relapses (group II). The average patient age was 37.4 (range: 27–58) among the group of patients with RRMS and 40.6 (range: 34–51) among the patients in the SPMS group.

The baseline EDSS scores ranged from 3 to 6.5 (mean 4.6) in the first group and from 3.5 to 9 (mean 5.6) in the second group.

The average disease duration was 9.5 years in the first group and 15.6 in the second group. All patients clinically deteriorated during the year preceding the study, with at least a 1-point increase in EDSS score. All of the included patients had failed previous treatments with first-line therapies (glatiramer acetate, interferon-b) or with second- and third-line therapies (fingolimod, natalizumab, azathioprine, and methotrexate) in different combinations.

Autologous MSCs were extracted from adipose tissue at the time of enrollment, and the patients were then observed and examined every 3 months. The number of MSCs extracted from a single collection of adipose tissues exceeded the target of 12 × 10^6^ cells. Cell suspensions in a cryoprotectant were divided into portions of 4 × 10^6^ cells/dose and cryopreserved in the stem cell bank at −170°C until transplantation. The patients received a portion of cells intrathecally at the 3- and 6-month follow-ups. Most patients received the target dose.

*Clinical Outcome, Disability, and Relapses.* Clinical data were available for the 20 included patients.

Within the first 12 months, 18 patients did not exhibit a change in EDSS score and did not have relapses. Two patients from the RRMS group had relapses without a change in EDSS score; the MRI scans of these patients showed new Gd+ lesions (in one patient, the lesions were in the brain, and, in the second patient, one lesion was observed in the medulla oblongata and pons).

At the 18-month follow-up, EDSS scores had not changed in any of the patients from either group. Within 18 months of follow-up, 3 patients from the RRMS group had relapses without exhibiting progression in disability from baseline, and 7 patients showed “enticing” improvement on some of the exploratory efficacy measures, although no significant benefit of the treatment on EDSS scores was observed across the entire group. All 7 of these patients showed a slight improvement in terms of MSFC scores and certain other efficacy measures. Overall, there was no significant change in any of these measures, although the improvements observed in these patients persisted throughout the total observation period. Four patients were observed for 24 months, and their clinical status did not change over this period. At 18 months after ASC transplantation, the EDSS scores of two of the patients from the SPMS group were slightly below the baseline value, but not by 1 point.

Adverse events after the administration of ASCs were not observed in any of the patients at the 12-, 18-, and 24-month follow-ups (Table 1).

## 4. Discussion

There is a critical unmet need to develop therapies that enable repair in MS patients. Preclinical studies using the mouse EAE model showed that intrathecal autologous hematopoietic stem cell transplantation (AHCT) improved neurological function and that this effect was associated with the suppression of local inflammatory responses and the provision of trophic support for damaged cells at the lesion site. As a result, new MS therapeutic strategies characterized by intense immunosuppression followed by AHCT have been proposed in recent years [11].

The suppression of inflammation that occurs after AHCT may play a beneficial role in slowing down disease progression and could induce prolonged tolerance to self-antigens [12].

In recent years, over 800 MS cases worldwide have been reported to the Registry of the European Group for Blood and Marrow Transplantation (EBMT) as having received this treatment procedure. Many patients have been treated in phase I/II studies, and good results have been reported [13]. A beneficial effect of intense immunosuppressive chemotherapy and AHCT in the treatment of aggressive MS that is unresponsive to standard therapies was first observed several years ago. Later, a major suppressive effect on disease activity was noted based on brain MRI, and the procedure was associated with 3% mortality [11]. Over the next several years, studies in which AHCT was used as a rescue therapy for malignant forms of MS were reported [14]. The most impressive results in patients with the malignant form of MS that were treated with AHCT were observed on MRI [15]. Although this method of therapy appears to be very effective, especially for select MS patients, only one published prospective study has compared AHCT with conventional treatments [13]. In this study, AHT was shown to be significantly superior to MTX in reducing disease progression on MRI and the annual relapse rate (ARR): patients with severe cases of MS in the AHCT arm experienced 79% fewer new T2 lesions and a lower ARR compared to patients in the MTX arm.

Another study in which mesenchymal stem cell-neural progenitors, an autologous bone marrow-derived cell population with regenerative potential, were administered intrathecally also showed that this treatment was safe and well tolerated in MS patients and that no adverse events occurred [16].

Despite the short observation time, the results of our study suggest that ASC intrathecal therapy is safe for use in MS and slows disease progression. As a powerful anti-inflammatory and immunosuppressive treatment, this method may benefit patients with rapidly progressive MS. ASCs are an attractive candidate for cell-based therapies aimed at stopping and reversing the loss of myelin in MS, which is ultimately responsible for the progressive disability that is observed over time in the majority of patients. However, the study did not have sufficient power to detect a significant clinical benefit, likely due to the short observation time. We expect to observe greater posttreatment benefits during the next period of observation. Nevertheless, there are still many unknowns regarding how to best deliver cell-based therapies in MS, such as the best route of administration, the need for ex vivo manipulations, and the desired dosing levels and intervals. For this reason, we do not view it as a problem that these early studies did not show a dramatic clinical benefit.

Both in vivo animal experiments and clinical observations suggest that ASC treatment causes no adverse effects [10, 13] These cells, however, should not be transplanted into cancer patients because MSC may induce the formation of blood vessels and secrete cytokines which may stimulate existing tumors, although they do not convert into cancer cells. For that reason, the recruitment of SM patients must exclude these with coexisting cancer disease.

Our experience with autologous mesenchymal stem cell transplantation in MS raises questions about the acceptable balance between safety, efficacy, and convenience when treating patients with MS. The future importance of these therapies will thus reflect a trade-off between the associated benefits and risks. In our opinion, ASCs are an attractive candidate for cell-based therapies aimed at stopping and reversing myelin loss in MS, which is ultimately responsible for the progressive disability that is observed in most patients over time. In our opinion, ASC therapy is not recommended for all patients with MS and should be reserved for aggressive cases, for cases still in the inflammatory phase of the disease, and for the malignant form.

In conclusion, no difference in 18-month EDSS score changes was found between the groups of RR and SP MS patients. No progression occurred in any patients from either group at the end of the follow-up, the 18th month of observation. A closely watched one-sided prospective trial of ASCs for MS demonstrated that the treatment was safe and suggested that it may have helped some patients. There were no serious or severe adverse treatment-related effects of any kind. No other treatment-related adverse effects were observed in patients who received intrathecal infusions of autologous ASCs, and the study hit safety endpoints. Disease progression-free survival (PFS) was 18 months for the majority of patients and did not differ according to disease type, gender, conditioning, or EDSS score at transplantation.

## Figures and Tables

**Table 1:** Baseline demographic characteristics and disease history of patients with the relapsing-remitting and secondary progressive form of MS.

	RRMS	SPMS
Number of patients	13	7
Male/female	9/4	3/4
Age	37,4 (range: 27–58)	40,6 (range: 34–51)
Duration of disease (years)	9,5	15,6
Neurologic status, median (range) EDSS score	4,6 (range: 3–6,5)	5,6 (range: 3,5–9)
Relapses 12 months before treatment	1-2	1-2
MRI activity: single-dose gadolinium positive or new or enlarging T2 lesions Gd+ after 12 months of treatment	4	0

## References

[1] Pugliatti M., Rosati G., Carton H. (2006). The epidemiology of multiple sclerosis in Europe. *European Journal of Neurology*.

[2] Kingwell E., Marriott J. J., Jetté N. (2013). Incidence and prevalence of multiple sclerosis in Europe: a systematic review. *BMC Neurology*.

[3] Ramagopalan S. V., Knight J. C., Ebers G. C. (2009). Multiple sclerosis and the major histocompatibility complex. *Current Opinion in Neurology*.

[4] Boster A., Edan G., Frohman E. (2008). Intense immunosuppression in patients with rapidly worsening multiple sclerosis: treatment guidelines for the clinician. *The Lancet Neurology*.

[5] Forostyak S., Homola A., Turnovcova K., Svitil P., Jendelova P., Sykova E. (2014). Intrathecal delivery of mesenchymal stromal cells protects the structure of altered Perineuronal nets in SOD1 rats and amends the course of ALS. *STEM CELLS*.

[6] Constantin G., Marconi S., Rossi B. (2009). Adipose-derived mesenchymal stem cells ameliorate chronic experimental autoimmune encephalomyelitis. *Stem Cells*.

[7] Klinker M. W., Wei C. (2015). Mesenchymal stem cells in the treatment of inflammatory and autoimmune diseases in experimental animal models. *World Journal of Stem Cells*.

[8] Donders R., Vanheusden M., Bogie J. F. J. (2015). Human Wharton’s jelly-derived stem cells display immunomodulatory properties and transiently improve rat experimental autoimmune encephalomyelitis. *Cell Transplantation*.

[9] Kim K. S., Lee H. J., An J. (2014). Transplantation of human adipose tissue-derived stem cells delays clinical onset and prolongs life span in ALS mouse model. *Cell Transplantation*.

[10] Bowles A. C., Scruggs B. A., Bunnell B. A. (2014). Mesenchymal stem cell-based therapy in a mouse model of Experimental Autoimmune Encephalomyelitis (EAE). *Methods in Molecular Biology*.

[11] Fassas A., Nash R. (2004). Multiple sclerosis. *Best Practice & Research: Clinical Haematology*.

[12] Mancardi G. L., Murialdo A., Rossi P. (2005). Autologous stem cell transplantation as rescue therapy in malignant forms of multiple sclerosis. *Multiple Sclerosis*.

[13] Mancardi G. L., Sormani M. P., Gualandi F. (2015). Autologous hematopoietic stem cell transplantation in multiple sclerosis: a phase II trial. *Neurology*.

[14] Portaccio E., Amato M. P., Siracusa G. (2007). Autologous hematopoietic stem cell transplantation for very active relapsing-remitting multiple sclerosis: report of two cases. *Multiple Sclerosis*.

[15] Kimiskidis V. K., Sakellari I., Tsimourtou V. (2008). Autologous stem-cell transplantation in malignant multiple sclerosis: a case with a favorable long-term outcome. *Multiple Sclerosis*.

[16] Harris V., Vyshkina T., Chirls S. (2015). *Interim Analysis of a Phase I Clinical Trial Investigating Intrathecal Administration of Mesenchymal Stem Cell-Neural Progenitors in Multiple Sclerosis*.

